# Heme-Oxygenase-1 Attenuates Oxidative Functions of Antigen Presenting Cells and Promotes Regulatory T Cell Differentiation during *Fasciola hepatica* Infection

**DOI:** 10.3390/antiox10121938

**Published:** 2021-12-03

**Authors:** Monique Costa, Valeria da Costa, Sofía Frigerio, María Florencia Festari, Mercedes Landeira, Santiago A. Rodríguez-Zraquia, Pablo Lores, Paula Carasi, Teresa Freire

**Affiliations:** Laboratorio de Inmunomodulación y Desarrollo de Vacunas, Departamento de Inmunobiología, Facultad de Medicina, Universidad de La República, Montevideo 11800, Uruguay; monique.scosta14@gmail.com (M.C.); valedacosta21@gmail.com (V.d.C.); frigeriosofia@gmail.com (S.F.); mffestari@gmail.com (M.F.F.); meche.land@gmail.com (M.L.); santiagorzq@gmail.com (S.A.R.-Z.); pablolores@hotmail.com (P.L.); paulacarasi@gmail.com (P.C.)

**Keywords:** helminth, heme-oxigenase-1, immunoregulation, antigen presenting cell, regulatory T cell, ROS/RNS

## Abstract

*Fasciola hepatica* is a fluke that infects livestock and humans causing fasciolosis, a zoonotic disease of increasing importance due to its worldwide distribution and high economic losses. The parasite regulates the host immune system by inducing a strong Th2 and regulatory T (Treg) cell immune response through mechanisms that might involve the expression or activity of heme-oxygenase-1 (HO-1), the rate-limiting enzyme in the catabolism of free heme that also has immunoregulatory and antioxidant properties. In this paper, we show that *F. hepatica*-infected mice upregulate HO-1 on peritoneal antigen-presenting cells (APC), which produce decreased levels of both reactive oxygen and nitrogen species (ROS/RNS). The presence of these cells was associated with increased levels of regulatory T cells (Tregs). Blocking the IL-10 receptor (IL-10R) during parasite infection demonstrated that the presence of splenic Tregs and peritoneal APC expressing HO-1 were both dependent on IL-10 activity. Furthermore, IL-10R neutralization as well as pharmacological treatment with the HO-1 inhibitor SnPP protected mice from parasite infection and allowed peritoneal APC to produce significantly higher ROS/RNS levels than those detected in cells from infected control mice. Finally, parasite infection carried out in gp91^phox^ knockout mice with inactive NADPH oxidase was associated with decreased levels of peritoneal HO-1^+^ cells and splenic Tregs, and partially protected mice from the hepatic damage induced by the parasite, revealing the complexity of the molecular mechanisms involving ROS production that participate in the complex pathology induced by this helminth. Altogether, these results contribute to the elucidation of the immunoregulatory and antioxidant role of HO-1 induced by *F. hepatica* in the host, providing alternative checkpoints that might control fasciolosis.

## 1. Introduction

Fasciolosis is a major parasitic disease of livestock caused by the trematode *Fasciola* spp. [1]. Nowadays, the number of infected people around the world is increasing, which makes fasciolosis an emerging zoonosis [1]. The World Health Organization (WHO) estimates that approximately 180 million are at risk of infection and 17 million people are infected, with a high prevalence in humans in Africa and South America [2]. Moreover, the economic losses caused by fasciolosis are estimated at around 3 billion US dollars per year due to livestock infection [1].

During infection, *Fasciola hepatica* modulates the host immune response characterized by the presence of regulatory dendritic cells (DC) [2,3,4,5], alternative activated macrophages [6], and an adaptive immune response characterized by Th2 and regulatory T cell (Treg)-associated cytokines [7,8,9,10]. Macrophages and DC comprise a highly heterogeneous myeloid cell population specialized in antigen presentation. DC play a crucial role in orchestrating the adaptive immune response by activating naïve T cells and inducing their differentiation into different effector T cells depending on the pathogen [11,12]. Furthermore, both macrophages and DC can promote inflammation by secreting pro-inflammatory cytokines and reactive oxygen or nitrogen species (ROS/RNS), although they can also inhibit inflammation through anti-inflammatory cytokine production [13,14,15]. Thus, they exhibit functional plasticity that enables them to adapt to various local conditions in order to restore homeostasis after inflammation [11,12,16].

Heme-oxygenase-1 (HO-1) is the inducible rate-limiting enzyme involved in the catabolism of free heme. It participates in several processes, by providing cytoprotection, protecting from heme-induced toxicity, and regulating the host inflammatory response [14,17]. In fact, HO-1 acts as a stress-responsive enzyme induced by the nuclear factor NF-E2-related factor 2 (NRF2), to provide defense against oxidative-induced injury during inflammatory processes [14]. HO-1 also limits the secretion of pro-inflammatory cytokines [18,19] and promotes anti-inflammatory cytokines [20,21], in a process triggered, at least in part, by ROS in macrophages, although its effects depend on the model of study [14]. In the same line, expression of HO-1 inhibits DC-maturation and the production of ROS [22]. In addition, HO-1 expression by DC induces the production of IL-10, an anti-inflammatory cytokine, that inhibits T-cell proliferation [23]. Additionally, IL-10-producing anti-inflammatory M2c macrophages express HO-1 [24], especially on those that express CD163, a hemoglobin scavenger receptor, that in fact mediates IL-10 production and HO-1 synthesis [25]. Therefore, HO-1 acts as a key mediator of anti-inflammatory pathways in both macrophages and DC.

Several reports demonstrate that HO-1 can have both beneficial and detrimental effects for the host immunity against different pathogens [26]. Furthermore, the antioxidant role of HO-1 in infectious diseases is still unclear, especially in helminth parasites. Interestingly, bilirubin, one of the enzymatic byproducts of HO-1, suppresses the killing of bacteria by reducing the neutrophil burst via its antioxidant activity [27]. In addition, HO-1 promotes bacteria survival inside macrophages by decreasing ROS production, as demonstrated for S*almonella typhimurium* [28] and *Mycobacterium abscessus* [29] via its ROS-diminishing properties. Recently, we showed that the anti-inflammatory effects of HO-1 induction are detrimental in *F. hepatica* infection, which is dominated by a Th2/Treg differentiation profile [17]. However, the antioxidant mechanisms induced by HO-1 during *F. hepatica* infection have not been approached so far.

Thus, in this work, we investigated the role of ROS/RNS production by myeloid HO-1^+^ cells during *F. hepatica* experimental infection in mice, and characterized the adaptive cell immune response. Our results indicate that HO-1 expression by myeloid cells during *F. hepatica* infection negatively correlates with the production of ROS or RNS and the increase of antioxidant molecules. Furthermore, the pharmacological inhibition of HO-1 by a well-characterized inhibitor of HO-1 enzymatic activity, tin protoporphyrin IX (SnPP), in *F. hepatica*-infected mice was associated with lower levels of Tregs, in a process that was mediated by IL-10 biological activity. However, the gene expression of the NADPH oxidase subunit gp91^phox^ was decreased when SnPP was administrated to infected mice. Moreover, parasite infection carried out in gp91^phox^ knockout mice with inactive NADPH oxidase showed decreased levels of peritoneal HO-1^+^ cells, splenic Tregs, and partially protected mice from the hepatic damage induced by the parasite, revealing that a more complex molecular mechanism involving ROS production participates in the intricate pathology induced by this helminth. Altogether, these results contribute to the elucidation of the immunoregulatory and antioxidant role of HO-1 during *F. hepatica* infection, providing interesting molecular checkpoints that might control fasciolosis.

## 2. Materials and Methods

### 2.1. Mice

Female BALB/c mice (six- to eight-week-old) were purchased from DILAVE Laboratories (Uruguay). Gp91^phox-^ knockout (B6.129S-Cybbtm1Din/J) mice were purchased from Jackson Laboratory (USA) and maintained at UATE, Institut Pasteur Montevideo. Six to eight BALB/c, gp91^phox-^, and C57BL/6 littermates (controls for gp91^phox-^ mice) were used per experiment. Animals were kept in the animal house (URBE, School of Medicine, UdelaR, Uruguay), with water and food supplied *ad libitum*. Mouse handling, care, and experiments were carried out in compliance with institutional guidelines and regulations from the National Committee on Animal Research (Comisión Nacional de Experimentación Animal, CNEA, https://www.cnea.gub.uy/, accessed on 12 November 2021, National Law 18.611, Uruguay). Procedures involving animals were approved by the Universidad de la República’s Committee on Animal Research (Comisión Honoraria de Experimentación Animal, CHEA Protocol Number 07153-000817-18).

### 2.2. Parasite Protein Extract (FhTE)

FhTE was prepared from live adult flukes obtained from infected bovines. Flukes were washed for 1 h at 37 °C with phosphate buffered saline (PBS), pH 7.4, sonicated, and then centrifugated at 40,000× *g* for 60 min [4,30]. Finally, protein lysates were dialyzed against PBS. FhTE protein concentration was measured using the bicinchoninic acid assay (Sigma, St. Louis, MO, USA). In order to remove endotoxin contamination, FhTE was applied to a column containing endotoxin-removing gel (detoxi-gel, Pierce Biotechnology, Waltham, MA, USA), and endotoxin levels were quantified using the Limulus Amebocyte Lysate kit Pyrochrome (Associates of Cape Cod, East Falmouth, MA, USA) and found to be lower than 0.05 EU/mL. Furthermore, at the used concentrations, FhTE did not induce the production of pro-inflammatory cytokines such as IL-12 or IL-6 [4,30]. The concentration of *F. hepatica* extracts used in the in vitro experiments did not modify cell viability, as evaluated by the MTT (2-[4,5-dimethyl-2-thiazolyl]-3,5-diphenyl-2H-tetrazolium bromide) assay [4,17,30].

### 2.3. F. hepatica Infections

#### 2.3.1. Parasite Infections, Animal Treatments, and Sample Obtention

The infection was achieved by orally administrating 10 *F. hepatica* metacercariae (Montevideo, Uruguay) per mouse. Mice were bled and peritoneal exudate cells (PECs), spleens, and livers were removed after 1, 8, 15, and 21 days post-infection (dpi), while non-infected animals were used as controls. Each experimental group contained at least four mice. PECs and hepatic leukocytes were obtained as already described [31]. Red cells were lysed with ammonium chloride potassium buffer.

HO-1 activity was inhibited using SnPP (40 mg/kg), and vehicle (PBS, 200 μL) was used as a control. The SnPP dose was within a range of doses used in previous works [32,33]. Mice received intraperitoneal injections of SnPP 1 day before infection, 1 day after infection, and every 4 days until the end of the experimental protocol (between 19 and 21 dpi). When gp91^phox-^ and non-infected littermates were used (*n* = 6–8/group), infections were performed in the same conditions as previously described. In order to neutralize IL-10 receptor (IL-10R), BALB/c mice (*n* = 6–8/group) were intraperitoneally injected with 15 μg of monoclonal rat IgG2a anti-IL-10R (clone 1B1.3A from BioXcell, Lebanon, NH, USA) or an isotype-matched control antibody (clone HRPN from BioXcell, Lebanon, NH, USA), the day before and after infection with *F. hepatica* and every 3 days until animal sacrifice at 20 dpi. Blood samples were obtained, and PECs, spleens, and livers were removed. The infection severity was assessed with a defined clinical score according to the following parameters: presence or absence of peritoneal hemorrhage, presence of macroscopic liver damage and splenomegaly, and the amount of cell content in the peritoneal cavity [17], where the minimum score was 0 and the maximum was 10. The alanine aminotransferase (ALT) activity in sera was used to quantify liver damage, determined with a commercial kit (Spinreact, Girona, Spain) according to the manufacturer’s instructions.

#### 2.3.2. Proliferation Assay and Cell Culture

Splenocytes (0.5 × 10^6^/well) from infected mice or uninfected naïve mice (control group) were cultured for 5 days at 37 °C and 5% CO_2_, in RPMI-1640 with 400 µg/mL of glutamine (Capricorn, Ebsdorfergrund, Germany) complete medium containing 10% heat-inactivated fetal bovine serum (FBS, Capricorn Scientific, Ebsdorfergrund, Germany), 50 mM of 2-mercaptoethanol, 100 U/mL of penicillin, and 0.1 mg/mL of streptomycin (Merk, Sigma-Aldrich, St. Louis, MO, USA) in the presence or absence of FhTE (75 µg/mL), as previously described [30]. An IFNγ-specific sandwich ELISA assay (Biolegend, San Diego, CA, USA) was used to quantify IFNγ levels in culture supernatants.

RAW264.7 macrophages were cultured at 0.5 × 10^6^/mL in complete RPMI medium in the presence or absence of the HO-1 inductor (CoPP) and inhibitor (SnPP) (50 and 100 µM, respectively) or FhTE (75 µg/mL) overnight at 37 °C. Afterwards, the ROS/RNS production was determined as described in the following section.

### 2.4. Flow Cytometry

Cell suspensions from PECs, livers, and spleens were washed twice with PBS containing 2% FBS and 0.1% sodium azide (FACS buffer), stained with specific antibodies for 30 min at 4 °C as previously published [31]. The following antibodies were used: anti-Sirp⍺ (P-84), -CD11c (N418), -CD86 (GL1), CD8 (53-6.7), -Siglec-F (E50-2440), -F4/80 (BM8), -CD11b (M1/70), -CD40 (HM40-3), -CD80 (16-10A1), and I-A/I-E (M5/114.15.2). Expression of FoxP3, HO-1, and IL-10 was analyzed by intracellular staining. Cells in which IL-10 was analyzed were incubated with Brefeldin-A for 6 h at 37 °C and phorbol myristate acetate (PMA, 200 nM) (Merk, Sigma-Aldrich, USA). After two washes with FACS buffer, cells were incubated with the following antibodies: anti-CD3 (17A2), -CD4 (RM4-5), -CD8 (53-6.7), or -F4/80 (BM8). After permeabilization with Cytofix and Perm wash buffers (Biolegend, USA), cells were incubated with IL-10 (JES5-1E3), FoxP3 (MF14), and HO-1 (clone ab13248 from Abcam, Waltham, MA, USA) specific antibodies. ROS/RNS produced by F4/80^+^ cells were determined with 2′,7′-dichlorofluorescein diacetate (DCFDA, Merk, Kenilworth, NJ, USA) probe, a fluorogenic dye that is oxidized into the fluorescent 2′,7′-dichlorofluorescein. Briefly, cells were incubated in PBS for 30 min at 37 °C with DCFDA, washed with FACS buffer, and fluorescence was measured in a flow cytometer. Analyses were performed using a BD Accuri C6 Plus cytometer and software (BD-Biosciences). Antibodies were obtained from Biolegend (USA). Analyses were performed with Accuri C6 Plus software.

### 2.5. Determination of Oxidative and Antioxidative Genes by qRT-PCR

*Nrf2*, *catalase*, glutathione peroxidase (*gpx*) 1 and 2, superoxide dismutase (*sod*) 1 and 2, and NADPH-oxidase subunits p47^phox^ and gp91^phox^ mRNA levels were detected using the Eco real-time PCR System (Illumina, San Diego, CA, USA) and Fast SYBR^®^ Green Master Mix (Applied Biosystems, Waltham, MA, USA). ARN purification was performed with Tri-Reagent (Merk, Kenilworth, NJ, USA) of PECs obtained from BALB/c mice at 20 dpi, SnPP-treated or untreated, as previously described [17]. Standard amplification conditions were 10 min at 95 °C, 40 thermal cycles of 15 s at 95 °C, 30 s at 60 °C, and 30 s at 72 °C, with a final extension of 10 min at 72 °C. The following primers were used: *nrf2*-F: 5′-CAGCATGTTACGTGATGAGG-3′, *nrf2*-R: 5′-GCTCAGAAAAGGCTCCATCC-3′, *gpx1*-F: 5′- GGGACTACACCGAGATGAACGA-3′, *gpx1*-R: 5′-ACCATTCACTTCGCACTTCTCA-3′, *gpx2*-F: 5′-GAGGAACAACTACCCGGGACTA-3′, *gpx2-R*: 5′-ACCCCCAGGTCGGACATACT-3′, *sod1*-F: 5′-TGGGTTCCACGTCCATCAGTA-3′, *sod1*-R: 5′-ACCGTCCTT TCCAGCAGTCA-3′, *sod2*-F: 5′-ATTAACGCGCAGATCATGCA-3′, *sod2*-R: 5′-TGTCCCCCACCATTGAACTT-3′, *catalase*-F: 5′-GCGTCCAGTGCGCTGTAGA-3′, c*atalase*-R: 5′-TCAGGGTGGACGTCAGTGAA-3′, *p47phox*-F: 5′-GAGGCGGAGGATCCGG-3′, *p47phox*-R: 5′-TCTTCAACAGCAGCGTACGC-3′, *gp91phox*-F: 5′-CCAGTGAAGATGTGTTCAGCT-3′, *gp91phox*-R: 5′-GCACAGCCAGTAGAAGTAGA-3′, *gapdh*-F: 5′-ATGACATCAAGAAGGTGGTGAAG-3′, *gapdh*-R: 5′-TCCTTGGAGGCCATGTAGG-3′. Results were expressed as the ratio between each gene under study and GAPDH expression. Relative gene expression levels were calculated using the 2^−ΔΔCT^ method and normalized to GAPDH. All reactions were performed with at least five biological replicates.

### 2.6. Statistical Analysis

Results of the experiments were expressed as mean ± SEM. GraphPad Prism version 6.04 for Windows (GraphPad Software, San Diego, CA, USA) was used to perform statistical analyses. Results were analyzed using one-way ANOVA followed by Tukey’s test, or the two-tailed Student’s *t*-test, depending on the experiment. Significant differences shown by an asterisk were considered when *p* < 0.05.

## 3. Results

### 3.1. HO-1 Expression in F4/80^+^ Peritoneal Cells Inversely Correlate with ROS/RNS Production

**Figure 1:**
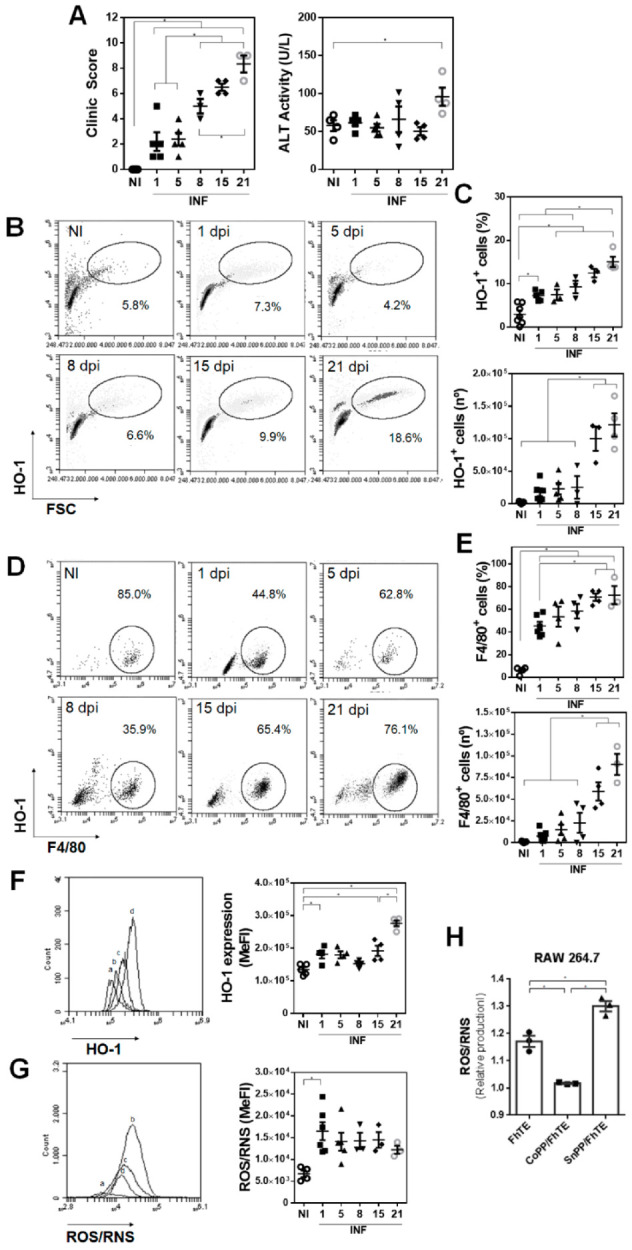
HO-1 expression in F4/80^+^ peritoneal cells inversely correlates with ROS/RNS production. Mice (*n* = 4–6) were infected with 10 metacercariae and sacrificed at 1, 8, 15, and 21 dpi. Non-infected mice were used as control (NI). (**A**) Clinical signs including hemorrhage, splenomegaly, and macroscopic liver damage were assessed to evaluate the severity of the disease [17]. ALT activity in sera was used as a marker of liver damage. (**B**) Analysis by flow cytometry of HO-1^+^ cells in PEC from infected and control (NI) mice. (**C**) Frequency and cell number of HO-1^+^ cells in the peritoneal cavity of infected and control (NI) animals by flow cytometry. (**D**) Analysis by flow cytometry of F4/80^+^ HO-1^+^ cells in PEC from infected and control (NI) mice. (**E**) Frequency and cell number of F4/80^+^ in the peritoneal cavity of infected and control (NI) animals by flow cytometry. (**F**) HO-1 expression in F4/80^+^ in the peritoneal cavity of infected and control (NI) mice. Letters on histograms correspond as follows: a: NI, b: 1 dpi, c: 15 dpi, d: 21 dpi. Median fluorescence intensity is shown (MeFI) in the plot. (**G**) ROS/RNS quantification in F4/80^+^ in the peritoneal cavity of infected and control (NI) mice using the DCFDA probe by flow cytometry. Letters on histograms correspond as follows: a: NI, b: 1 dpi, c: 15 dpi, d: 21 dpi. Median fluorescence intensity is shown (MeFI) in the plot. (**H**) Murine RAW264.7 macrophages were cultured in the presence of 75 µg/mL of FhTE or CoPP (100 µM/mL) and SnPP (50 µM/mL) overnight at 37 °C. Then, cells were collected and incubated for 30 min at 37 °C in PBS with the DCFDA probe and analyzed by flow cytometry. The RNS/ROS levels are shown as the ratio between FhTE/medium (FhTE), CoPP + FhTE/CoPP (CoPP/FhTE), and SnPP + FhTE/SnPP (SnPP/FhTE). Representative experiments are shown. Asterisks indicate significant differences with *p* < 0.05, performed by one-way ANOVA followed by Tukey’s test with multiple comparisons.

In order to confirm the recruitment of PECs expressing HO-1 to the peritoneum of *F. hepatica*-infected mice, we identified HO-1*^+^* cells by flow cytometry at different time points of the infection. As seen in Figure 1A, the clinical score increased upon infection, although ALT in serum significantly increased only after 21 dpi, demonstrating liver dysfunction. In addition, HO-1*^+^* cells significantly increased in the peritoneal cavity during the infection (Figure 1B,C and Supplementary Figure S1). These cells were mainly composed by F4/80*^+^* cells (Figure 1D,E and Supplementary Figure S1), and their increase also correlated with the advanced stages of the infection (after 15 dpi). The expression of HO-1 in F4/80*^+^* cells slightly increased after 1 dpi, while it considerably increased during the infection (Figure 1F). On the other hand, the production of ROS/RNS was significantly increased only at 1 dpi, and decreased during infection (Figure 1G), suggesting that the expression of HO-1 in peritoneal F4/80*^+^* cells inversely correlated with the production of ROS/RNS. In order to provide more evidence in this regard, we incubated RAW 264.7 macrophages with parasite components (FhTE) in the presence of CoPP or SnPP, and analyzed the production of ROS/RNS by these cells. FhTE slightly increased the production of ROS/RNS, while CoPP and SnPP significantly decreased and increased the production of ROS/RNS by FhTE-treated macrophages, respectively (Figure 1H). Of note, FhTE *per se* induced ROS/RNS expression, which could be the result of an active respiratory burst, such as that seen in F4/80^+^ cells from PECs of infected mice at 1 dpi (Figure 1G). Altogether, these results might indicate that *F. hepatica* induces the expression of HO-1 in F4/80*^+^* cells recruited to the peritoneum, inhibiting ROS/RNS production during the course of the infection.

### 3.2. The Presence of Peritoneal HO-1^+^ Cells Associates with Increased Splenic CD4^+^ CD25^+^ and CD8^+^ CD25^+^ Cells during Infection

**Figure 2:**
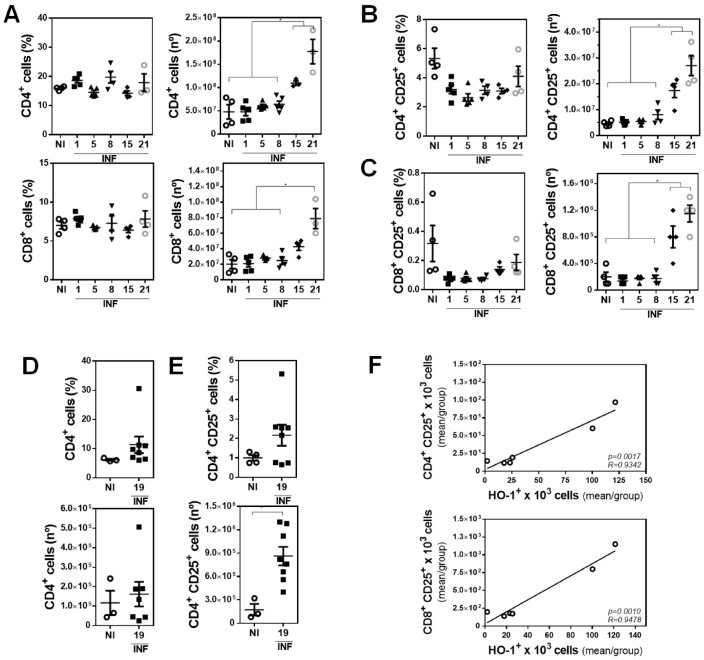
Peritoneal HO-1^+^ cells correlate with increased splenic CD4^+^ CD25^+^ and CD8^+^ CD25^+^ cells during *F. hepatica* infection. Mice (*n* = 4–8/group) were infected with 10 metacercariae and sacrificed at 1, 8, 15, and 21 dpi. Non-infected mice were used as control (NI). (**A**) Frequency and cell number of splenic CD4^+^ or CD8^+^ cells from infected and control (NI) mice. (**B**,**C**) Frequency and cell number of splenic CD4^+^ CD25^+^ (**B**) or CD8^+^ CD25^+^ (**C**) cells from infected and control (NI) mice. (**D**) Frequency and cell number of hepatic CD3^+^ CD4^+^ cells from infected and control (NI) mice. (**E**) Frequency and cell number of hepatic CD4^+^ CD25^+^ cells from infected and control (NI) mice (right). (**F**) Splenic CD4^+^ CD25^+^ or CD8^+^ CD25^+^ cell number in function of the number of peritoneal HO-1^+^ cells in NI and infected mice. The mean of CD4^+^ CD25^+^ (in Figure 2B), CD8 CD25^+^ (in Figure 2C), and HO-1^+^ cells (in Figure 1C) was plotted. Gate analyses by flow cytometry are shown in Supplementary Figures S2 and S3. The results shown represent one experiment. Asterisks indicate significant differences with *p* < 0.05, performed by one-way ANOVA followed by Tukey’s test with multiple comparisons.

Considering that HO-1 can induce regulatory T cells [34,35,36,37], we analyzed the presence of both CD4*^+^* and CD8*^+^* cells in spleens of infected mice. Although we could not find any significant differences between the percentage of CD4*^+^* and CD8*^+^* cells during the infection, we did observe that they significantly increased in number after 15 dpi (Figure 2A and Supplementary Figure S2). We also analyzed the presence of splenic CD25*^+^* CD4*^+^* (Figure 2B and Supplementary Figure S2) or CD8^+^ (Figure 2C and Supplementary Figure S2) T cells. Again, no significant differences were found in the percentage of these cells during the infection, while their number significantly increased after 15 dpi. Finally, we analyzed the presence of CD4*^+^* T cells in livers from infected animals and did not find any difference in their percentage nor their number (Figure 2D and Supplementary Figure S3). However, the number, but not the frequency, of hepatic CD25*^+^* CD4*^+^* T cells was increased in advanced infected mice (Figure 2E and Supplementary Figure S3). Further analyses demonstrated that the number of splenic CD25*^+^* CD4*^+^* and CD25*^+^* CD8*^+^*cells positively correlated with the number of peritoneal HO1*^+^* cells (Figure 2F).

### 3.3. HO-1 Activity Decreases the Production of ROS/RNS by F4/80^+^ Cells and Correlates with an Increase of Splenic Regulatory CD4^+^ T Cells Induced by F. hepatica Infection

**Figure 3:**
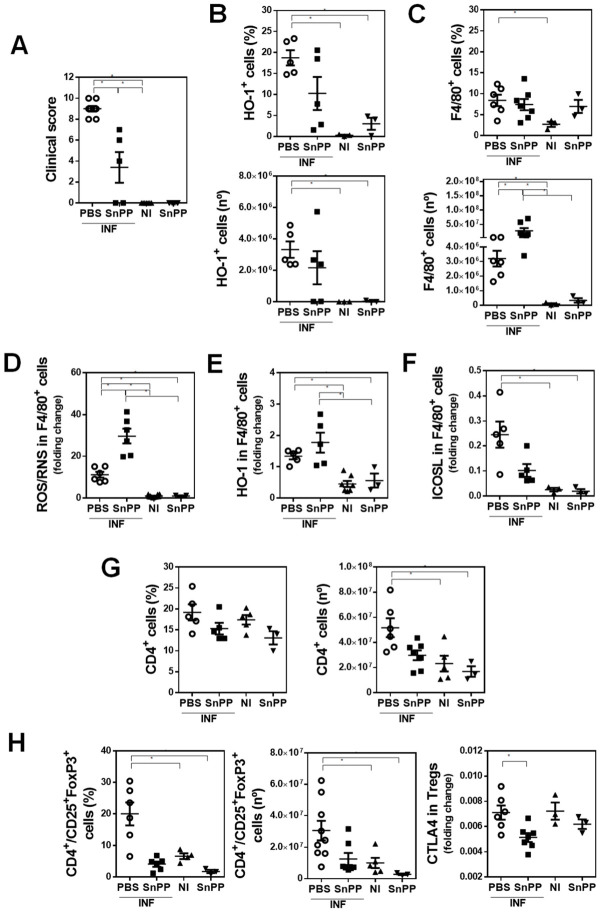
The HO-1 pharmacological inhibitor SnPP decreases the production of ROS/RNS by peritoneal F4/80^+^ cells and correlates with an increase of splenic FoxP3^+^CD25^+^/CD4^+^ T cells induced by *F. hepatica* infection. Mice were injected with SnPP (40 mg/kg) or vehicle (PBS) one day before infection and every 4 days until the end of the experimental protocol. Mice (*n* = 5/group) were infected with 10 metacercariae (day 0) and sacrificed at 20 dpi. Non-infected (NI) mice (*n* = 3/group) both treated and untreated with SnPP were used as control. (**A**) Clinical signs of infected mice were analyzed to assess disease severity [17]. (**B**) Frequency and cell number of HO-1^+^ cells in the peritoneal cavity of SnPP-treated or untreated infected and control (NI) animals by flow cytometry. (**C**) Frequency and cell number of F4/80^+^ cells in the peritoneal cavity of SnPP-treated or untreated infected and control (NI) animals by flow cytometry. (**D**) ROS/RNS quantification in F4/80^+^ cells of the peritoneal cavity of SnPP-treated or untreated infected and control (NI) mice using the DCFDA probe by flow cytometry. HO-1 (**E**) and ICOSL (**F**) expression in F4/80^+^ cells in the peritoneal cavity of SnPP-treated or untreated infected and control (NI) mice. (**G**) Splenic CD4^+^ T cell frequency and number from SnPP-treated or untreated infected and control (NI) mice. (**H**) Splenic CD4^+^/CD25^+^FoxpP3^+^ cell frequency and number from SnPP-treated or untreated infected and control (NI) mice (left). CTLA4 expression in splenic CD4^+^/CD25^+^FoxpP3^+^ cells from SnPP-treated or untreated infected and control (NI) mice. Gate analyses by flow cytometry are shown in Supplementary Figures S2 and S4. Results obtained for one representative experiment are shown. Asterisks indicate significant differences with *p* < 0.05, performed by one-way ANOVA followed by Tukey’s test with multiple comparisons.

In order to evaluate whether HO-1 interferes with the production of ROS/RNS, we treated *F. hepatica*-infected mice with the HO-1 inhibitor SnPP. SnPP treatment was associated with a decrease in the clinical signs of infected mice (Figure 3A). In addition, SnPP treatment of infected mice abrogated the increase of HO-1*^+^* cells, both in frequency and number, induced by the infection, since no significant difference was found in infected mice with respect to the control group with SnPP treatment (Figure 3B and Supplementary Figure S4). Surprisingly, a significant increase in F4/80*^+^* cell number, but not frequency, was found in both SnPP-treated and non-treated infected mice (Figure 3C and Supplementary Figure S4). Indeed, the F4/80^+^ cell number was higher in SnPP-treated infected mice. Nevertheless, F4/80*^+^* cells of SnPP-treated infected mice produced higher levels of ROS/RNS than control infected mice (Figure 3D), although they expressed similar levels of HO-1 (Figure 3E). Of note, the expression of ICOSL in peritoneal F4/80*^+^* cells of infected mice was significantly reduced with SnPP treatment (Figure 3F). Lastly, SnPP treatment during *F. hepatica* infection did not induce an increase in the number of splenic CD4^+^ T cells (Figure 3G) or CD4*^+^*/CD25*^+^* T cells (Figure 3H), although these cells expressed higher levels of CTLA4 in the absence of SnPP treatment (Figure 3H). Altogether, these results suggest that HO-1 activity inhibited by SnPP decreases the production of ROS/RNS during fasciolosis and correlates with an increase of splenic regulatory CD4*^+^* T cells in a process that might involve ICOSL in antigen-presenting cells or CTLA4 expression in Tregs.

**Figure 4:**
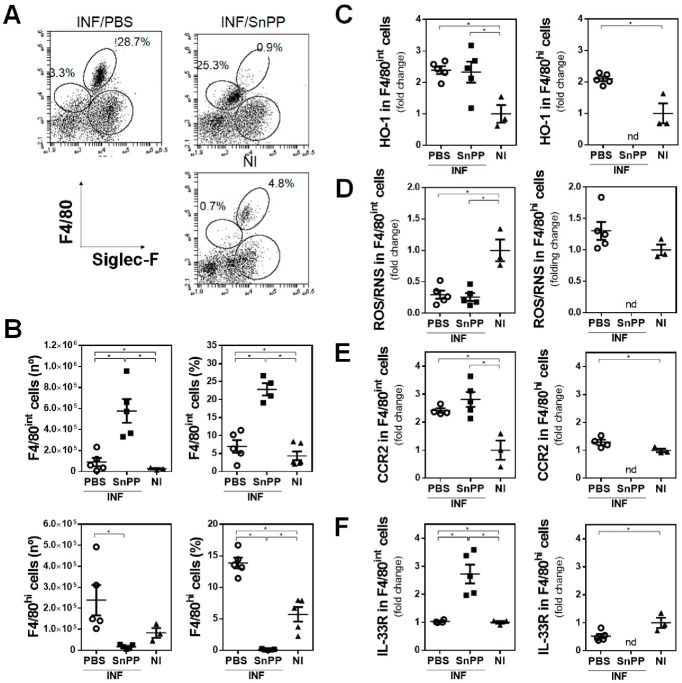
Analyses of peritoneal F4/80^+^ cells at the early events of *F. hepatica* infection. Mice (*n* = 5/group) were injected with SnPP (40 mg/kg) or vehicle (PBS) one day before infection, infected with 10 metacercariae (day 0), and sacrificed at 1 dpi. Non-infected (NI) mice (*n* = 3/group) both treated and untreated with SnPP were used as a control. (**A**) Analysis of F4/80^int^ and F4/80^hi^ cells in PEC from SnPP-treated or untreated infected and control (NI) mice by flow cytometry. (**B**) Frequency and cell number of F4/80^int^ (upper plots) or F4/80^hi^ (lower plots) cells in the peritoneal cavity of SnPP-treated or untreated infected and control (NI) animals. HO-1 (**C**), ROS/RNS (**D**), CCR2 (**E**), and IL-33R (**F**) expression in peritoneal F4/80^int^ and F4/80^hi^ cells by flow cytometry. “nd” means none detected, since barely any F4/80^hi^ cells in SnPP-treated infected mice were detected. Median fluorescence intensity is shown (MeFI) in the plot. Gate analyses by flow cytometry are shown in (**A**) and Supplementary Figure S1. Representative experiments are shown. Asterisks indicate significant differences with *p* < 0.05, performed by one-way ANOVA followed by Tukey’s test with multiple comparisons.

To complement these results, we evaluated whether the inhibition of HO-1 by SnPP treatment affected the recruitment or the phenotypical characteristics of peritoneal F4/80*^+^* cells at the early stages of *F. hepatica* infection. To this end, we analyzed F4/80*^+^* cells in the peritoneal cavity of SnPP-treated mice at 1 dpi and compared them with both non-treated infected and control mice. We observed the presence of two different cell populations according to F4/80 expression (Figure 4A and Supplementary Figure S1). SnPP treatment increased both the frequency and number of F4/80^int^ cells, while it reduced F4/80^hi^ cells in the peritoneal cavity of infected mice at 1 dpi (Figure 4B). Nevertheless, F4/80^int^ cells expressed similar levels of HO-1 (Figure 4C) and ROS/RNS (Figure 4D) in F4/80^int^ cells regardless of SnPP treatment. Interestingly, peritoneal F4/80^int^ cells expressed higher levels of CCR2 (Figure 4E), while only those from SnPP-treated infected mice expressed significantly increased levels of IL-33R (Figure 4F), which could be related to the initiation of an early immune response against the parasite. Thus, these results suggest that the presence of F4/80^int^ IL-33R^+^ cells in the peritoneum is induced by SnPP treatment, which in turn protects mice from infection.

### 3.4. The Inhibition of HO-1 Activity by SnPP Controls the Gene Expression of Antioxidant Molecules

**Figure 5:**
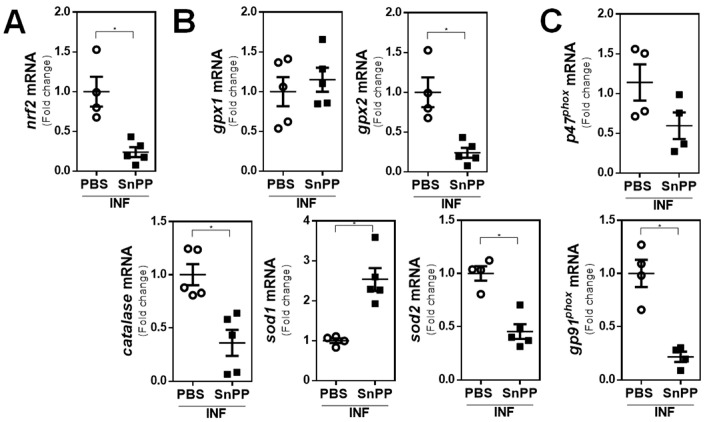
mRNA levels of antioxidative and oxidative genes in the infection by *F. hepatica*. Mice (*n* = 5/group) were injected with SnPP (40 mg/kg) or PBS 1 day before infection, infected with 10 metacercariae (day 0), and sacrificed at 20 dpi. *nrf2* (**A**), *catalase, gpx1, gpx2, sod1, sod2* (**B**), and *p47phox* and *gp91phox* (**C**) gene expression in PECs from SnPP-treated and control infected mice evaluated by qRT-PCR. mRNA levels were analyzed by qRT-PCR with respect to *gapdh* expression in PECs from SnPP-treated and control infected mice (PBS). Results were compared to the infected (control) group of mice and represented as the ratio between gene expression in SnPP-treated and control mice. Asterisks indicate significant differences with *p* < 0.05, performed by the Student’s *t*-test.

To deeply analyze the relationship between HO-1 expression in peritoneal cells induced by *F. hepatica* infection with the production of ROS/RNS, the gene expression of different molecules involved in the oxidative and antioxidative responses was evaluated. PECs of SnPP-treated infected mice were characterized by a significant decrease in the mRNA levels of the transcription factor *nrf2* (Figure 5A). Moreover, the SnPP-induced decrease in *nrf2* gene expression levels was associated with decreased mRNA levels in the antioxidant enzymes *catalase,* glutathione peroxidase 2 (*gpx2*), and superoxide dismutase 2 (*sod2*) (Figure 5B). However, no differences were found in the gene expression of *gpx1*, while an increase in *sod1* expression was observed (Figure 5B). Finally, an unexpected decrease in the mRNA levels of the NADPH oxidase subunits *gp91^phox^* and *p47^phox^* was observed with SnPP treatment of infected mice (Figure 5C).

### 3.5. Deficiency of Functional NADPH Oxidase Partially Protects Mice from Liver Damage Induced by F. hepatica and Limits the Production of IL-10

**Figure 6:**
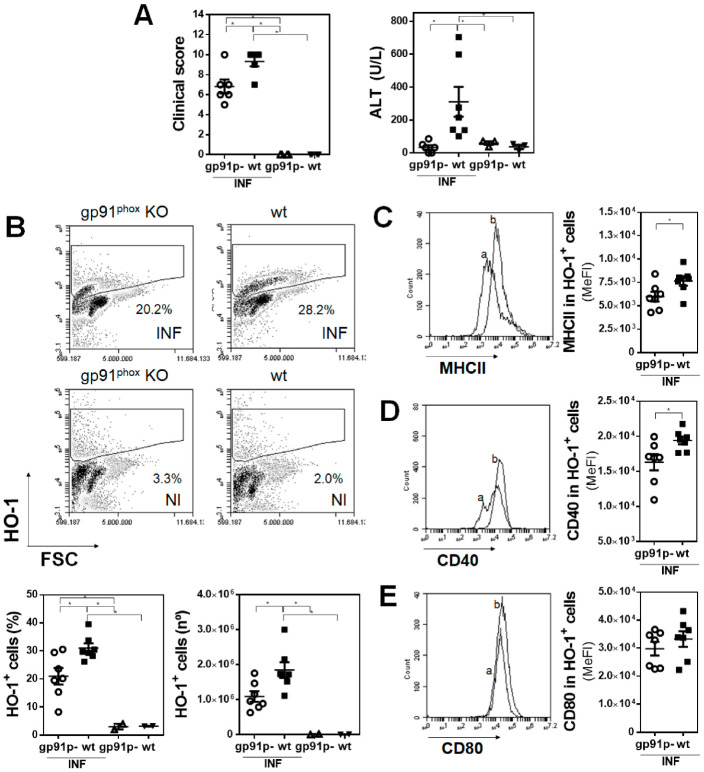
Gp91^phox^ (gp91p-) knockout (*n* = 6–8/group) and littermate control (*n* = 2/group) mice were infected with 10 metacercariae and sacrificed at 20 dpi. Non-infected mice were used as a control. (**A**) Clinical signs analyzed to assess disease severity [17]. Quantification of liver damage by ALT activity in sera. (**B**) Flow cytometry analysis of HO-1^+^ in PECs from gp91^phox^ knockout mice and littermate controls (upper panel). (**C**) Frequency and cell number of HO-1^+^ cells in the peritoneal cavity of infected and non-infected animals by flow cytometry (lower panel). MHCII (**C**), CD40 (**D**), and CD80 (**E**) expression in HO-1^+^ cells in the peritoneal cavity of infected and control mice. Letters on histograms correspond as follows: a: infected gp91^phox^, b: infected wildtype littermates. Median fluorescence intensity is shown (MeFI) in the plot. Asterisks indicate significant differences with *p* < 0.05, performed by one-way ANOVA followed by Tukey’s test with multiple comparisons.

**Figure 7:**
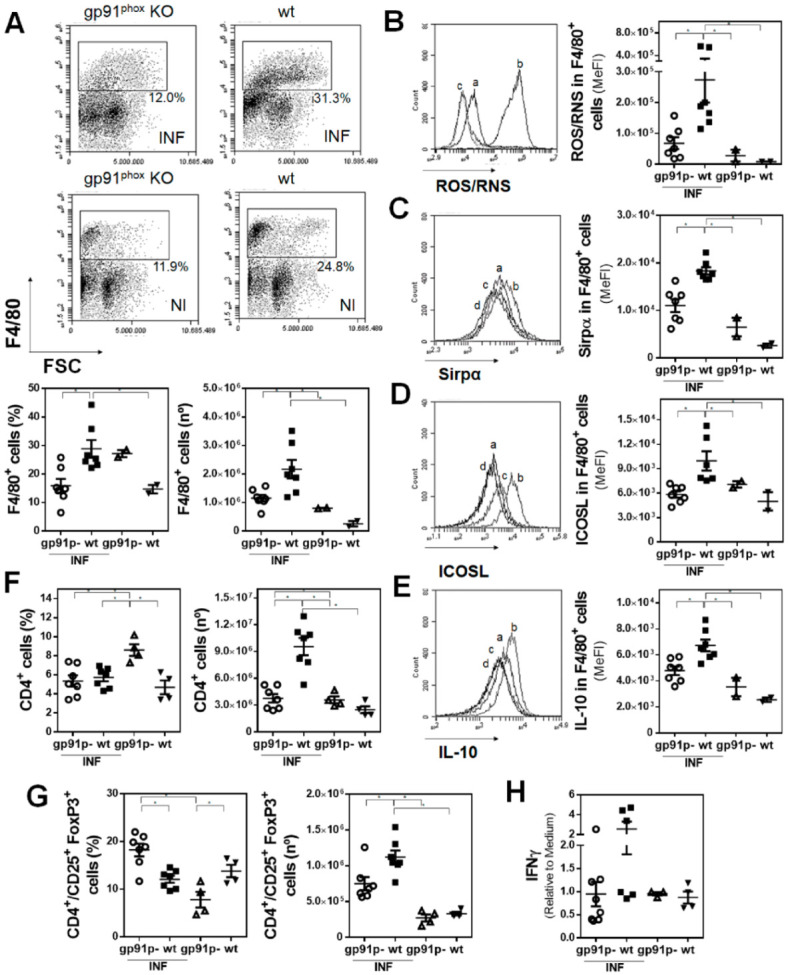
Peritoneal F4/80^+^ cells from wildtype infected mice present a regulatory-like phenotype. Gp91^phox^ (gp91p-) knockout (*n* = 7–8/group) and littermate control (*n* = 2–4/group) mice were infected with 10 metacercariae and sacrificed at 20 dpi. Non-infected mice were used as a control. (**A**) Flow cytometry analysis of F4/80^+^ cells in PEC from gp91^phox^ knockout mice and littermate controls (upper panel). Frequency and cell number of F4/80^+^ cells in the peritoneal cavity of infected and non-infected animals by flow cytometry (lower plots). ROS/RNS (**B**), Sirpα (**C**), ICOSL (**D**), and IL-10 (**E**) expression in F4/80^+^ cells in the peritoneal cavity of infected and uninfected mice. Letters on histograms correspond as follows: a: infected gp91^phox^, b: infected littermates, c: uninfected gp91^phox^, and d: uninfected littermates. Median fluorescence intensity is shown (MeFI) in the plot. (**F**) Frequency and cell number of splenic CD4^+^ cells from infected and non-infected mice. (**G**) Frequency and cell number of splenic CD4^+/^CD25^+^ Foxp3^+^ cells from infected and non-infected mice. Gate analyses by flow cytometry are shown in (**A**) and Supplementary Figure S1. (**H**) IFNγ levels in culture supernatants of splenocyte proliferation assay cultured with FhTE for 5 days at 37 °C. The results shown represent one experiment. Asterisks indicate significant differences with *p* < 0.05, performed by one-way ANOVA followed by Tukey’s test with multiple comparisons.

Considering the fact that SnPP treatment protected mice from parasite infection and that reduced levels of *gp91^phox^* mRNA were found in PECs from infected mice, we analyzed the infection in gp91^phox^ knockout mice. gp91^phox^ deficiency reduced the clinical signs and liver damage induced by *F. hepatica* infection (Figure 6A and Supplementary Figure S5). This partial protection was associated with a lower increase of HO-1*^+^* peritoneal cells, both in frequency and number (Figure 6B). Moreover, HO-1^+^ cells from gp91^phox^ knockout infected mice expressed lower levels of MHCII (Figure 6C) and CD40 (Figure 6D), but not CD80 (Figure 6E), than those from wildtype mice, indicating that NADPH oxidase may play a role both in the immune response and the pathogenesis induced by *F. hepatica* infection. Further characterization of the peritoneal cells from these mice indicated that the increase of F4/80*^+^* cells was abrogated in the absence of gp91^phox^ (Figure 7A), and as expected, very low levels of ROS/RNS produced by these cells (Figure 7B). Additionally, these cells expressed lower levels of Sirpα (Figure 7C), ICOSL (Figure 7D), and IL-10 (Figure 7E). Finally, lower numbers of CD4*^+^* (Figure 7F and Supplementary Figure S6) and CD4*^+^*/CD25*^+^*FoxP3*^+^* Tregs (Figure 7G and Supplementary Figure S6) were found in gp91^phox^ knockout infected mice with respect to wildtype mice. However, no significant differences in the production of IFNγ by splenocytes stimulated with parasite components (FhTE) between gp91^phox^ knockout and wildtype infected mice were detected (Figure 7H).

### 3.6. IL-10 Signaling Is Crucial for HO-1 Expression in F. hepatica-Infected Mice

**Figure 8:**
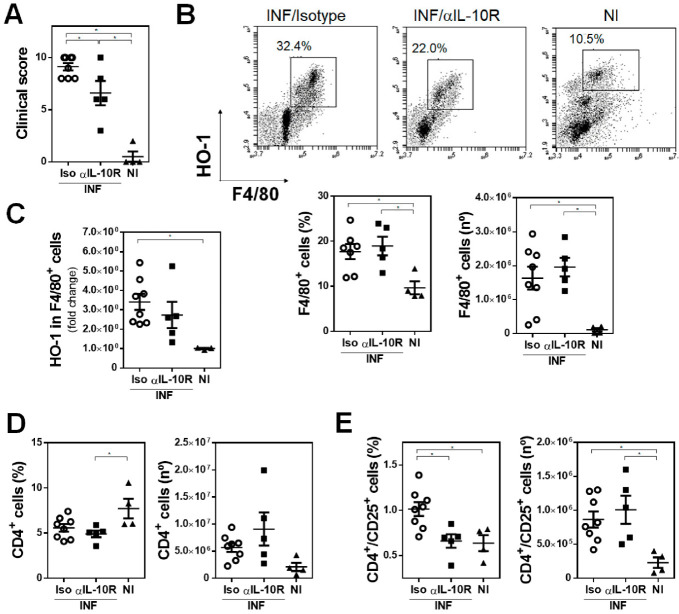
IL-10 signaling is essential for HO-1 expression in *F. hepatica*-infected mice. Fifteen µg of monoclonal rat IgG_2a_ anti-IL-10R antibody was administrated by intraperitoneal injection the day before and after infection with *F. hepatica* and every 3 days until sacrifice (*n* = 5–8/group). The control group (*n* = 4/group) received an isotype control antibody. At day 20 post-infection, animals were sacrificed and splenocytes were analyzed by flow cytometry. (**A**) Clinical signs were analyzed to assess disease severity [17]. (**B**) Analysis by flow cytometry of F4/80^+^ cells in PEC from infected and non-infected (NI) mice showing frequency and number of F4/80^+^ cells in PECs. (**C**) HO-1 expression in F4/80^+^ cells in the peritoneal cavity of infected and uninfected mice. (**D**) Frequency and cell number of CD4^+^ T cells in spleens from infected and non-infected (NI) mice. (**E**) Frequency and cell number of CD4^+^ CD25^+^ T cells in spleens from infected and non-infected (NI) mice. Gate analyses by flow cytometry are shown in (**B**) and Supplementary Figure S7. Representative results of one representative are shown. Asterisks indicate significant differences with *p* < 0.05, performed by one-way ANOVA followed by Tukey’s test with multiple comparisons.

Considering the fact that IL-10 induces HO-1 expression that can favor the production of IL-10, and that IL-10 is crucial for Treg differentiation [36], we analyzed whether there was a relationship between IL-10 signaling and HO-1 expression during *F. hepatica* infection. To this end, we treated infected mice with a neutralizing antibody of IL-10 receptor (IL-10R). The results demonstrate that IL-10R blocking reduced the clinical signs associated with parasite infection (Figure 8A). Although the recruitment of F4/80*^+^* cells in the peritoneum of infected mice was not affected by IL-10R neutralization (Figure 8B), it abrogated the elevated expression of HO-1 induced by *F. hepatica* infection (Figure 8C). Interestingly, IL-10R neutralization reduced the frequency, but not the number, of CD4*^+^* (Figure 8D and Supplementary Figure S7) and CD4*^+^*CD25*^+^* (Figure 8E and Supplementary Figure S7) T cells in the spleens of infected animals. Altogether, these results indicate that IL-10 signaling is essential for HO-1 expression of F4/80*^+^* cells during *F. hepatica* infection, likely affecting the differentiation of regulatory T cells.

## 4. Discussion

In this work, we have examined the cellular and molecular mechanisms that govern the expansion or differentiation of Tregs induced by HO-1^+^ cells in *F. hepatica* infection. We presented evidence showing that HO-1 activity results in decreased ROS/RNS production by F4/80^+^ antigen-presenting cells, thereby enhancing the pathological effects caused by *F. hepatica* and promoting parasite infection. Furthermore, apart from its antioxidant capacity, HO-1 has other functions, such as its immunoregulatory properties and controlling gene expression as a transcription factor [14,21,26,32,38]. Indeed, HO-1 inhibition promotes IFNγ- and NOS2-mediated control of *M. tuberculosis* infection in mice [39]. Furthermore, it has been previously reported that HO-1 has a role in suppressing pro-inflammatory Th1 immune responses in experimental colitis, and sickle cell alloimmunization has been reported, and it protects from atherosclerosis [40,41]. Finally, HO-1 can impair the immunity against other pathogens, such as *Plasmodium yoelii* [42].

**Figure 9:**
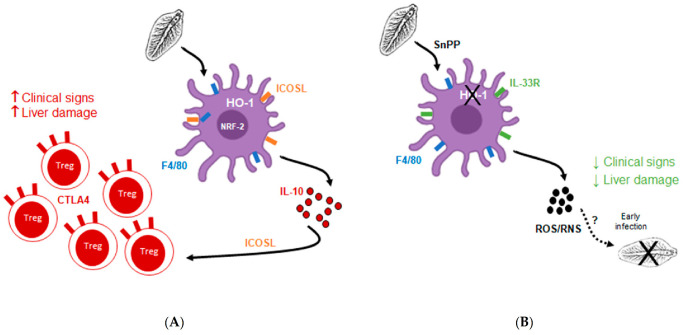
General hypothesis. (**A**) ICOSL^+^ F4/80^+^ APC express HO-1 induced by parasite infection that promotes IL-10 production and differentiation or expansion of CTLA4^+^ Tregs. (**B**) SnPP treatment inhibiting HO-1 activity in IL-33R^+^ F4/80^+^ APC allows ROS/RNS production, that induce parasite damage in early stages of the infection.

Indeed, we demonstrated that during *F. hepatica* experimental infection in mice, there is an increase in the expression of HO-1 in F4/80^+^ cells in the peritoneal cavity and it inversely correlates with ROS/RNS production. Furthermore, we demonstrated an association between the expression of HO-1 and the presence of putative Tregs in the spleens of infected animals (Figure 9A). These results were also confirmed when using the HO-1 inhibitor SnPP, which inhibits its enzymatic activity. At first sight, the inhibition of HO-1 activity by SnPP would suggest that its effects are caused by the heme-catabolizing activity rather than by its expression and function as a transcription factor. Indeed, F4/80^+^ peritoneal cells from SnPP-treated mice did not show a decrease in HO-1 expression, although a significant increase in ROS/RNS production was detected. SnPP is a metalloporphyrin that acts as a competitive inhibitor of HO-1 both in vitro and in vivo. Its efficiency can be explained by its higher binding affinity to HO-1/2 than to heme [43,44]. However, enzymatically inactive HO-1 can still mediate protection against hydrogen peroxide-induced toxicity, probably by promoting the gene expression of antioxidant proteins [14,45], although the mechanisms underlying these effects are still unclear. Thus, the possibility that HO-1 would act as transcription factor cannot be discarded, since the nuclear localization of HO-1 in F4/80^+^ cells derived from *F. hepatica*-infected mice with or without SnPP treatment was not investigated. Furthermore, it is unlikely that the protective outcome of SnPP treatment represents a direct effect on *F. hepatica*, since the degree of infection and pathological effects induced by the parasite were also related to an increase in Tregs, evidencing that HO-1 activity influences the host adaptive immunity in vivo. Indeed, our results indicate that the increase of the mRNA levels of *nrf2*, a transcription factor responsible for the regulation of cellular redox balance and protecting antioxidant responses [46,47], is accompanied by an increase in some antioxidant enzyme genes, demonstrating that the infection, HO-1, Tregs, and the Nrf2 master regulator comprise a complex axis of antioxidant and immunoregulatory properties in *F. hepatica* infection. However, the function of these enzymes should be determined in order to confirm their antioxidant role during *F. hepatica* infection. On the other hand, heme-activated murine macrophages have functional anti-inflammatory features that are dependent on the enzymatic activity of HO-1 [38]. Thus, the immunoregulatory and immunosuppressive properties of HO-1 together with its antioxidant properties demonstrate that its function during *F. hepatica* infection goes far beyond heme degradation itself.

The role of ROS/RNS in helminth parasite killing is still controversial. Some reports showed that the infection by *Strongyloides papillosus* induced an oxidative/nitrosative stress in sheep [48], although its effect on the parasite itself has not been demonstrated. On the other hand, *Schistosome* infection relates to an immense oxidative stress by the host that is not sufficient to control infection [49]. Further data demonstrated that excretory/secretory factors from *Mesocestoides corti* inhibit ROS-induced neutrophil extracellular traps, showing that the parasite could use this mechanism to attenuate the effects induced by ROS [50]. It should be highlighted, however, that although oxidative mechanisms are induced by helminth parasite infections, their detrimental role in the parasite itself as well as in the host surroundings is not well-established yet [51,52,53]. A recent report has demonstrated a high oxidative status in serum and liver in rabbits infected with *F. gigantica*, together with a decline in the SOD and catalase gene expression and enzyme activity in sera from infected animals [54], which is not in agreement with data from our work in *F. hepatica* experimentally infected mice. However, the authors came to the conclusion that the disruption of antioxidant and detoxification cascades by *F. gigantica* likely leads to the pathogenic response from the host [54].

It is worth noting that in our work, we used a DCFDA fluorescent probe that does not distinguish between ROS and RNS. Therefore, these studies should be complemented with others using ROS-specific probes such as DHE or specific inhibitors of nitric oxide production (such as L-Name). In order to analyze the ROS produced by NADPH-oxidase, we used, instead, gp91phox knockout mice. Interestingly, the fact that mice that are deficient in NADPH oxidase function, with a considerable decrease in ROS production, were partially protected against *F. hepatica* infection, suggests that the moment when ROS is produced by NADPH oxidase might be crucial to limit *F. hepatica*-induced damage (Figure 9B). Indeed, an exacerbated ROS production induced by a pro-inflammatory immune response can be detrimental to leukocyte cell function or viability and induced damage to the immune system [54]. Thus, a prolonged and not regulated production of ROS by F4/80^+^ cells could benefit the parasite, and not the host. Of note, these cells expressed higher levels of ICOSL and IL-10 than those from gp91^phox^ knockout mice, which could be associated with the differentiation or expansion of a higher number of splenic Tregs, which in turn express higher levels of CTLA4. Indeed, both ICOSL [55,56] and CTLA4 [57] are key mediators of Treg differentiation. In the same line, macrophages can suppress T cell responses and favor the expansion of Tregs [58]. Furthermore, ROS levels on T cell activation seem to be important, since small quantities of ROS result in antigen hypo-responsiveness, while high doses lead to oxidative stress-induced apoptosis [59]. Further analysis of the role of IL-10 produced by antigen-presenting cells in the differentiation or expansion of Tregs showed that IL-10 signaling is essential to increase HO-1 expression in peritoneal F4/80^+^ cells and likely the production of Tregs. Interestingly, it would seem that the parasite exploits the host defense mechanisms, on the one hand by recruiting HO-1^+^ cells with less antioxidative functions that produce IL-10, and on the other hand by in turn inducing the differentiation to Tregs. Nevertheless, the production of IL-10 by the host would also protect host cells in the acute pro-inflammatory immune response, caused either by damage induced by the parasite in the early state of the infection or by liver damage, at least in this experimental model. However, more experiments are needed in order to confirm these results, and to determine the role of ROS in the induction of Tregs and its relationship with IL-10.

One hypothesis that can explain these results might be the fact that ROS/RNS production is (partially) effective only during early stages of *F. hepatica* infection (Figure 9B). After ingestion of metacercariae by the mammalian host, juvenile flukes penetrate the host intestine wall and reach the liver through the peritoneal cavity between 4 and 6 days in livestock, although it is thought that it takes around 24 h in mice [3]. To further understand the early events that take place during *F. hepatica* infection in mice, we analyzed HO-1 expression and F4/80^+^ cell recruitment at 1 dpi, finding that two different populations expressing different levels of F4/80 are present in the peritoneum, and those elicited in SnPP-treated mice expressed higher levels of IL33R (Figure 9B). IL33 is an alarmin that participates in the type 2 innate immune response, promoting innate lymphoid cells type 2. However, during *Schisotosoma* infection, IL-33 seems to contribute to the development of pathology via the induction of type 2 innate lymphoid cells and alternative activation of macrophages, thus favoring the infection [60,61,62]. Therefore, the functions of IL-33 during *F. hepatica* infection in mice, and in particular the overexpression of its receptor in antigen-presenting cells at the early events of the infection, remain to be elucidated.

In conclusion, our work showed that HO-1 is a key molecule that favors *F. hepatica* infection, by which HO-1 could control ROS/RNS production and Treg differentiation and how the parasite elicits/triggers these mechanisms. Altogether, these results contribute to the elucidation of the immunoregulatory and antioxidant roles of HO-1 induced by *F. hepatica* in the host, providing interesting checkpoints that might control fasciolosis.

## Data Availability

Data is contained within the article and supplementary materials.

## Supplementary Materials

The following are available online at https://www.mdpi.com/article/10.3390/antiox10121938/s1, Figure S1: Gates used for flow cytometry analyses of PECs from infected and control mice corresponding to Figure 1B,D and Figure 4A; Figure S2: Gates used for flow cytometry analyses of splenocytes from infected and control mice corresponding to Figure 2A–C, Figure 3G,H; Figure S3: Gates used for flow cytometry analyses of hepatic leukocytes from infected and control mice corresponding to Figure 2D,E; Figure S4: Gates used for flow cytometry analyses of splenocytes from infected and control mice corresponding to Figure 3B,C; Figure S5: Representative images of livers from gp91^phox^ KO and C57BL/6 liitermates infected and control mice; Figure S6: Gates used for flow cytometry analyses of splenocytes from infected and control mice corresponding to Figure 7F,G; Figure S7: Gates used for flow cytometry analyses of splenocytes from IL 10R or isotype treated infected and control mice corresponding to Figure 8D,E.
