# Supplementary material for: Heme-Oxygenase-1 Attenuates Oxidative Functions of Antigen Presenting Cells and Promotes Regulatory T Cell Differentiation during Fasciola hepatica Infection
Source: Antioxidants (Basel). 2021 Dec 3;10(12):1938. doi: 10.3390/antiox10121938 (PMC8750899; doi:10.3390/antiox10121938)
Supplement: Supplementary file 1 [file antioxidants-10-01938-s001.zip › antioxidants-1426788-Supplementary.pdf]

Costa *et al.*

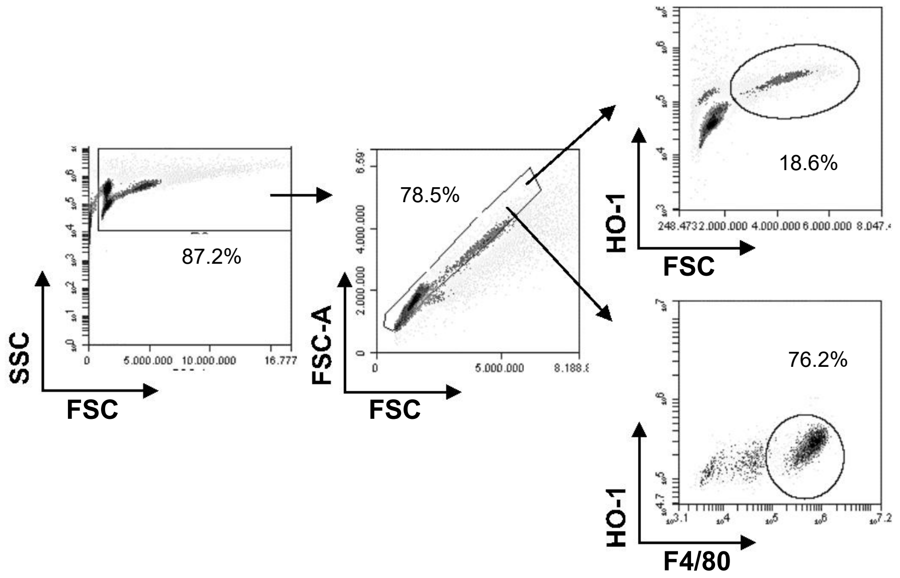

Supplementary Figure S1.
Gates used for flow cytometry analyses of PECs from infected and control mice corresponding to Figure 1B, 1D and 4A.

Supplementary Figure S2

Costa *et al.*

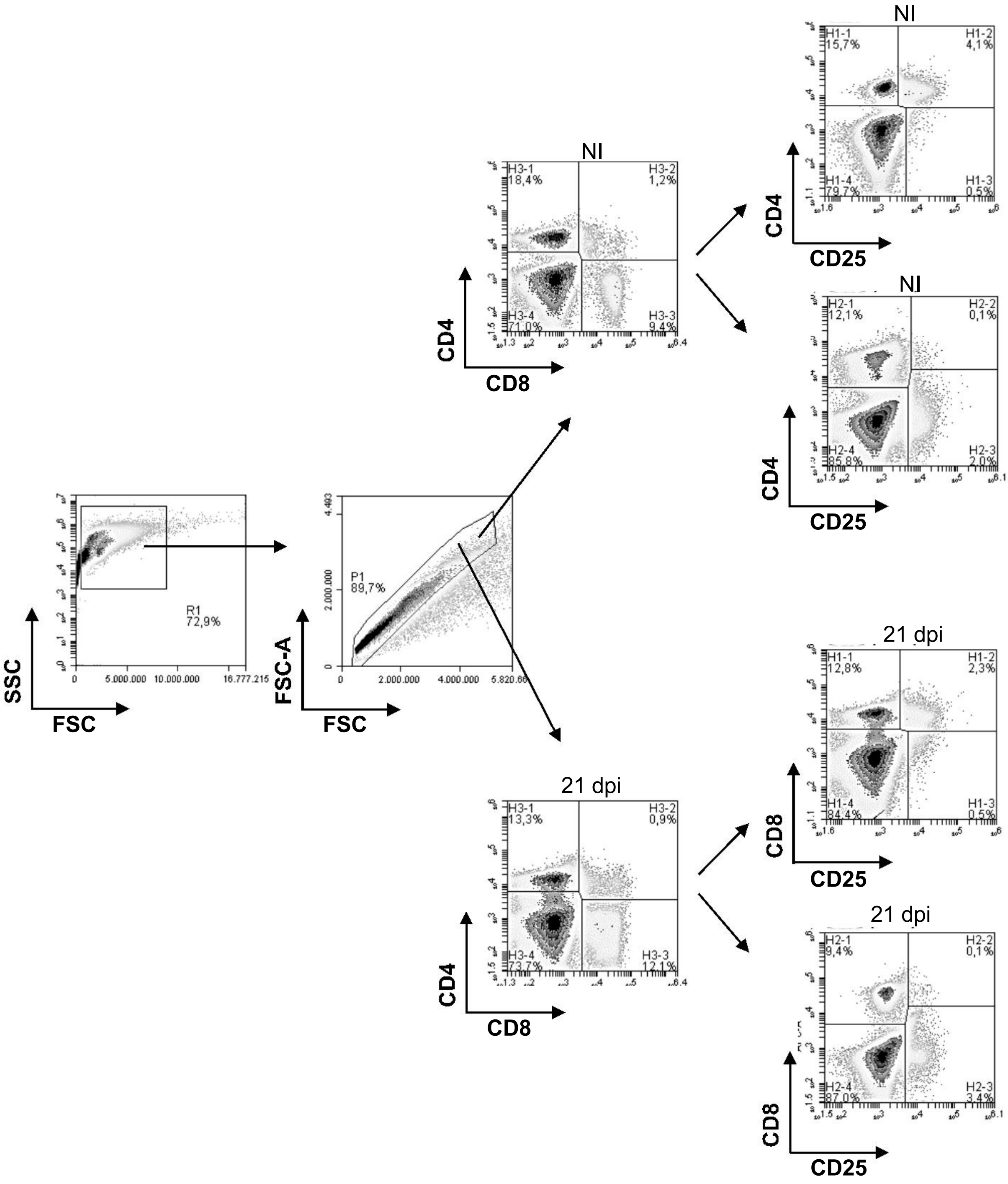

Supplementary Figure S2.
Gates used for flow cytometry analyses of splenocytes from infected and control mice corresponding to Figure 2A, 2B, 2C, 3G and 3H.

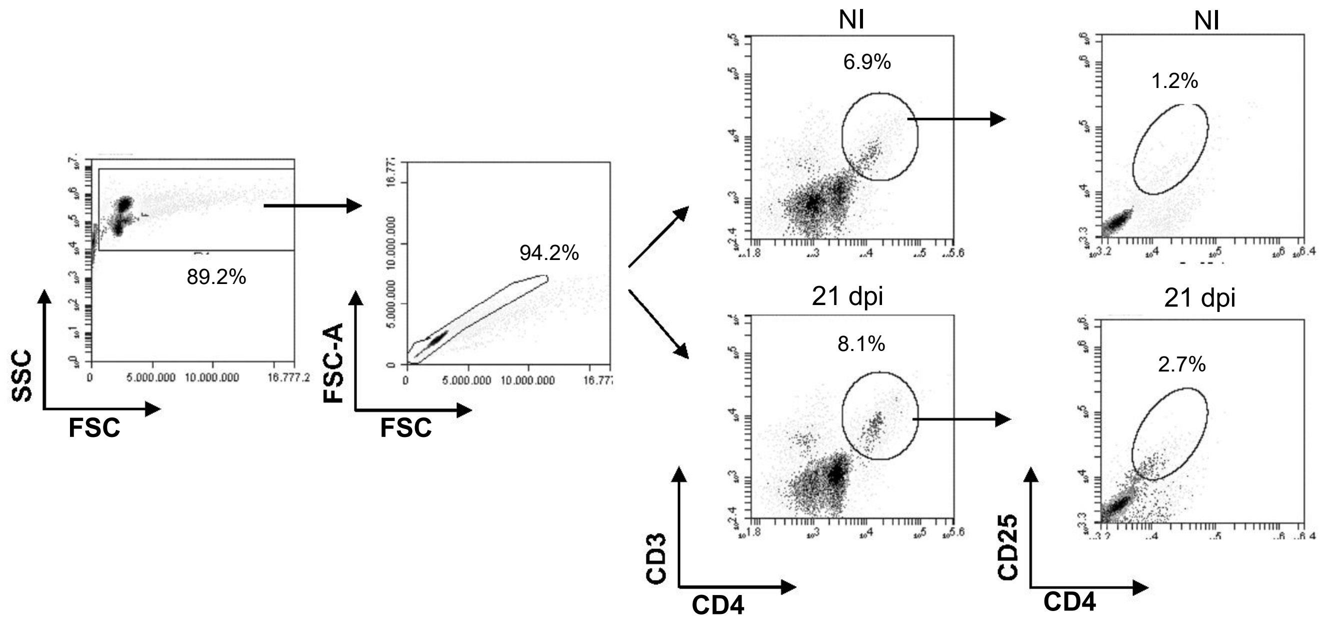

Supplementary Figure S3.
Gates used for flow cytometry analyses of hepatic leukocytes from infected and control mice corresponding to Figure 2D and 2E.

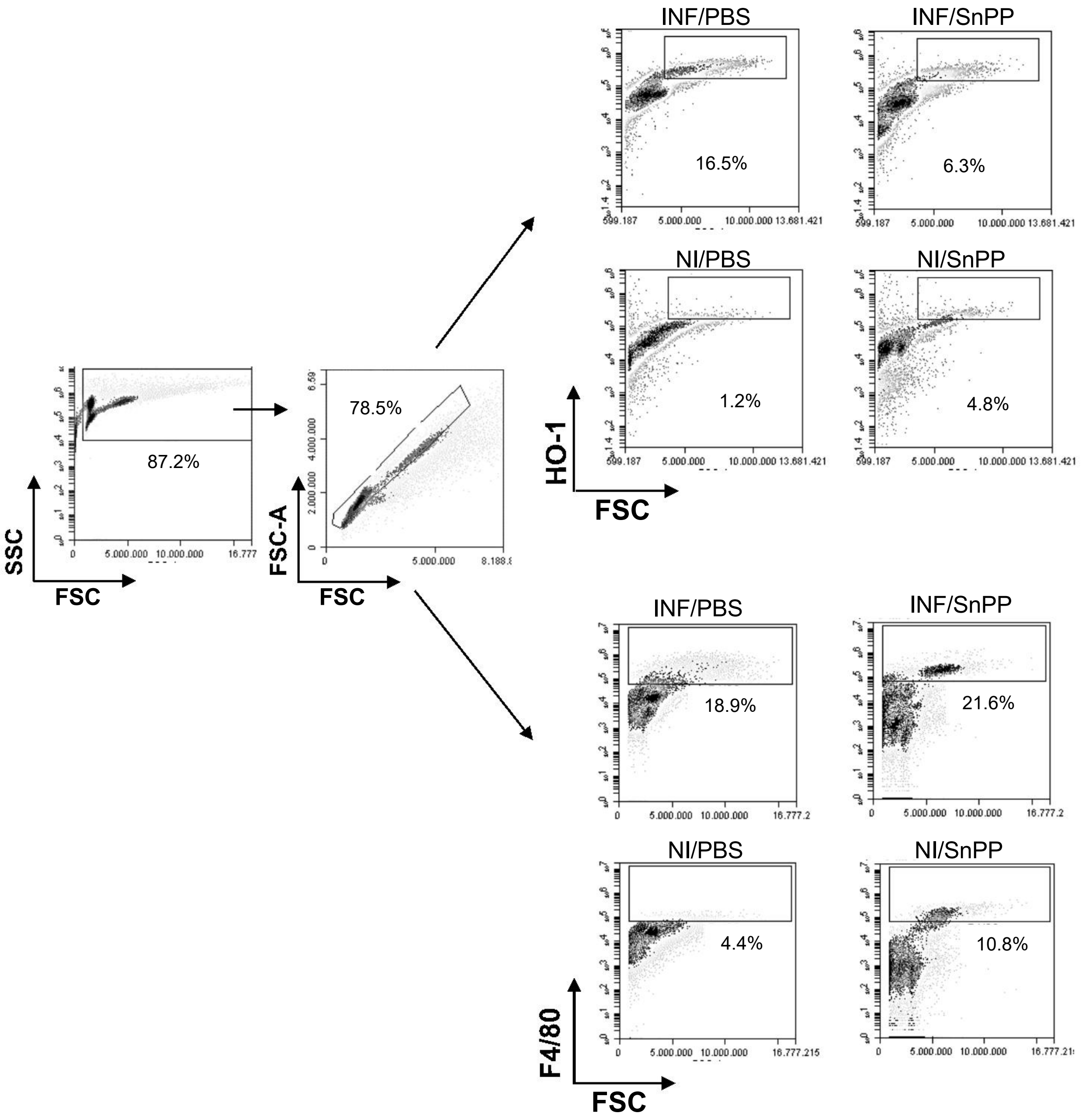

Supplementary Figure S4.
Gates used for flow cytometry analyses of splenocytes from infected and control mice corresponding to Figure 3B and 3C.

Costa *et al.*

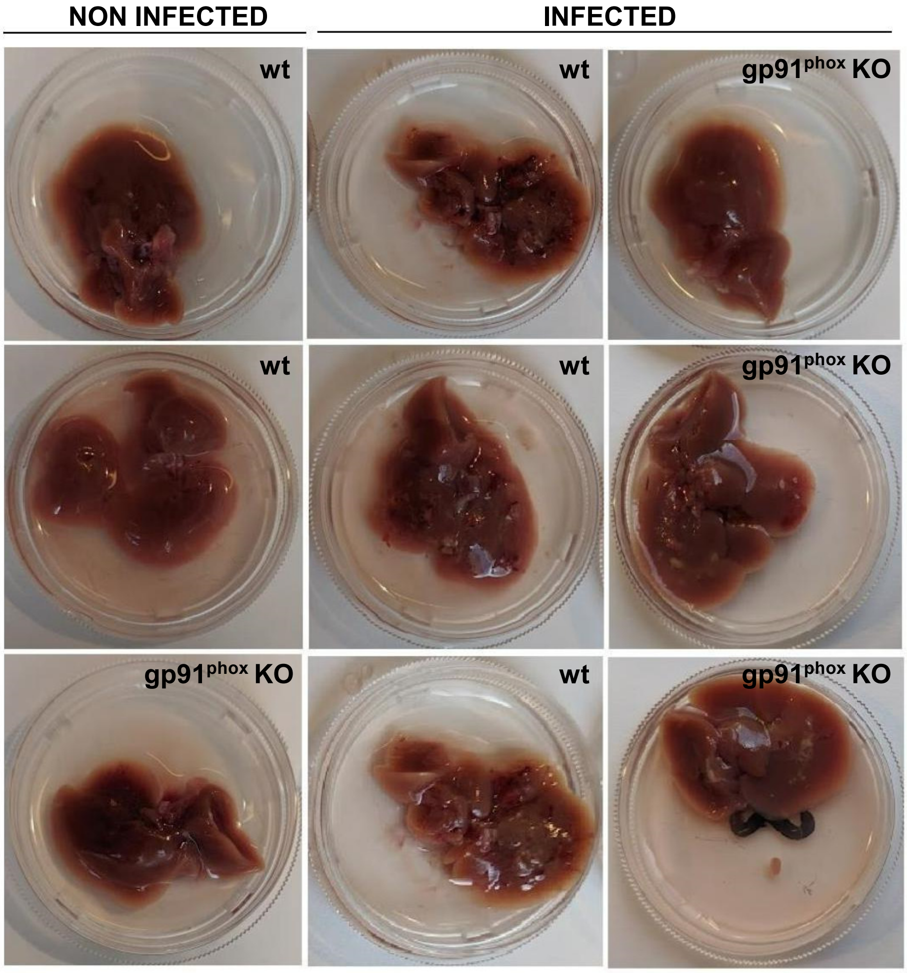

Supplementary Figure S5.
Representative images of livers from gp91^{phox} KO and C57BL/6 liitermates infected and control mice.

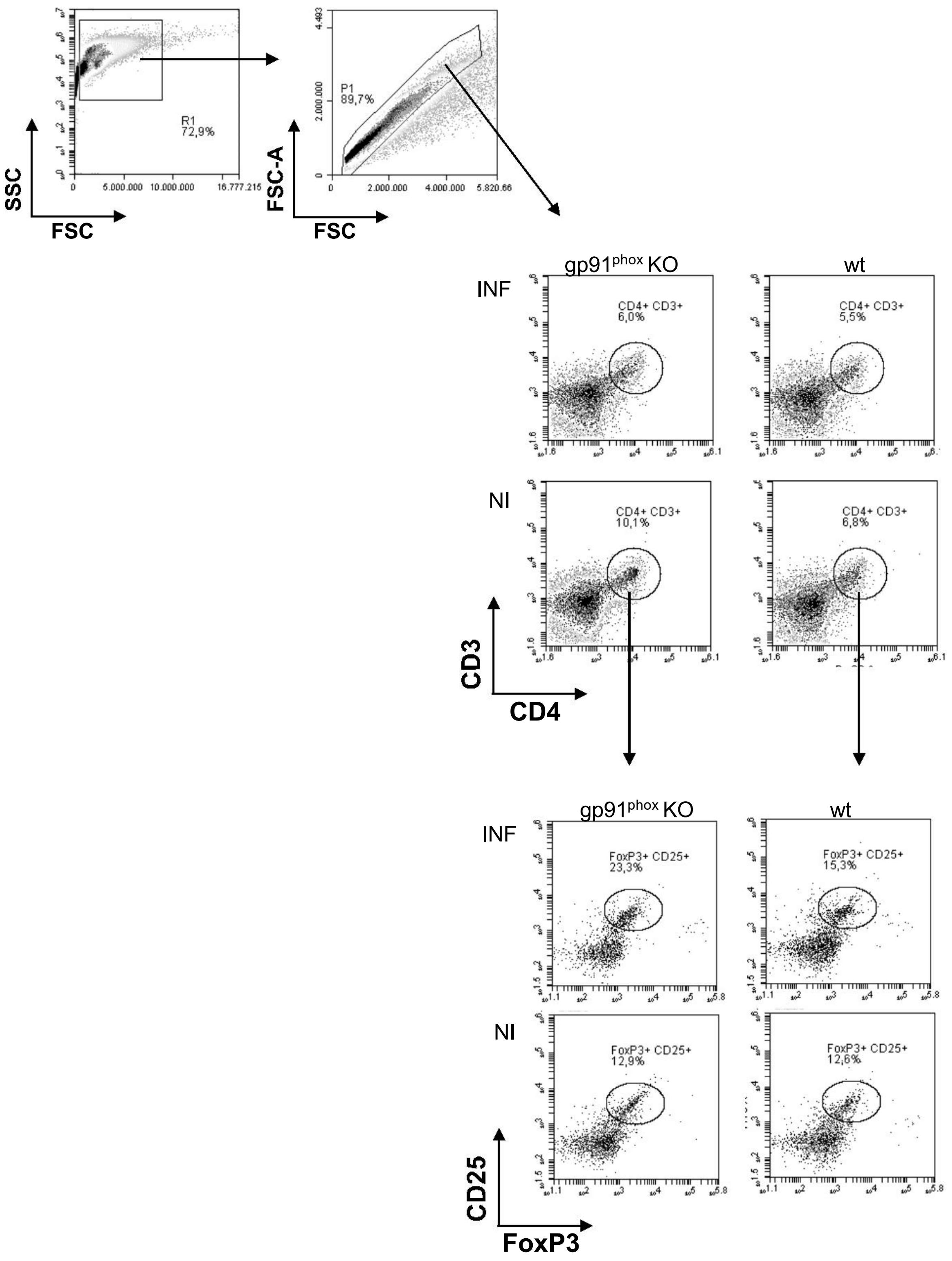

Supplementary Figure S6.
Gates used for flow cytometry analyses of splenocytes from infected and control mice corresponding to Figure 6F and 6G.

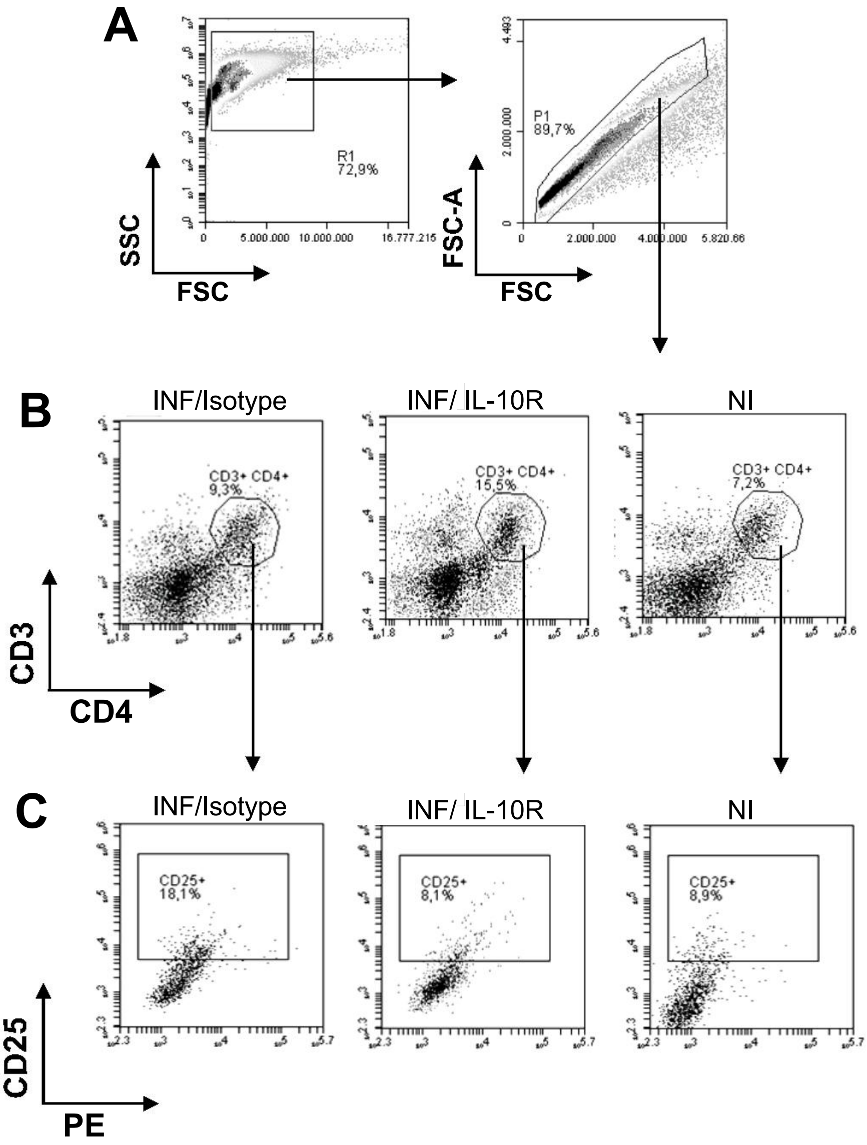

Supplementary Figure S7.
Gates used for flow cytometry analyses of splenocytes from IL-10R- or isotype-treated infected and control mice corresponding to Figure 8D and 8E.
